# Preoperative Inflammatory Ratios and Severe Intraoperative Hypoxemia During One-Lung Ventilation: A Prospective Observational Study

**DOI:** 10.3390/life16071057

**Published:** 2026-06-25

**Authors:** Irina Saplacan, Stefania Raluca Fodor, Bianca Liana Grigorescu, Manuela Rozalia Gabor, Oana Coman, Claudiu Puiac, Leonard Azamfirei

**Affiliations:** 1 Doctoral School of Medicine and Pharmacy, George Emil Palade University of Medicine, Pharmacy, Science and Technology of Târgu Mureș, 540142 Târgu Mureș, Romania; saplacanirina@yahoo.com (I.S.); manuela.gabor@umfst.ro (M.R.G.); 2 Department of Anesthesiology and Intensive Care Medicine, George Emil Palade University of Medicine, Pharmacy, Science and Technology of Târgu Mureș, 540103 Târgu Mureș, Romania; bianca.grigorescu@umfst.ro (B.L.G.); oana.coman@umfst.ro (O.C.); claudiu.puiac@umfst.ro (C.P.); leonard.azamfirei@gmail.com (L.A.); 3 Department of Economic Sciences (ED1), Faculty of Economics and Law, George Emil Palade University of Medicine, Pharmacy, Science and Technology of Târgu Mureș, 540142 Târgu Mureș, Romania

**Keywords:** one-lung ventilation, hypoxemia, thoracic surgery, inflammatory markers

## Abstract

(1) Background: One-lung ventilation (OLV) is frequently required during thoracic surgery, but hypoxemia remains a common intraoperative complication. Neutrophil-to-lymphocyte ratio (NLR) and platelet-to-lymphocyte ratio (PLR) have emerged as inexpensive inflammatory biomarkers, although their role in predicting hypoxemia during OLV remains unclear. This study evaluated the association between preoperative NLR, PLR, and severe intraoperative hypoxemia during OLV. (2) This interim analysis included 103 patients undergoing elective thoracic surgery with OLV in a prospective observational cohort. Severe hypoxemia was defined as PaO_2_/FiO_2_ < 100. Group comparisons were performed using Mann–Whitney U and chi-square/Fisher’s exact tests. Hierarchical logistic regression and ROC analysis were used to evaluate predictors and model performance. (3) Results: Preoperative PLR significantly improved the predictive performance of the clinical model for severe intraoperative hypoxemia, while NLR was not associated with the outcome. BMI remained an independent predictor of hypoxemia. (4) Conclusions: PLR improved the predictive performance of the clinical model, although its inverse association with hypoxemia should be interpreted cautiously. NLR was not associated with hypoxemia during OLV.

## 1. Introduction

One-lung ventilation (OLV) is frequently required during thoracic surgery and is the preferred technique for collapsing the operative lung while maintaining ventilation and oxygenation of the contralateral lung. By improving surgical exposure and reducing cross-contamination, OLV plays a central role in modern thoracic anesthesia [1].

A major challenge during OLV is hypoxemia, most commonly driven by ventilation–perfusion (V/Q) mismatch. In addition to OLV itself, V/Q mismatch is influenced by patient positioning, anesthesia, cardiac output, ventilation mode, pre-existing pulmonary conditions, and factors affecting hypoxic pulmonary vasoconstriction. Understanding these mechanisms is essential for optimal management [2,3].

OLV may also induce inflammatory and microvascular responses related to mechanical stress, atelectasis, re-expansion, oxygen exposure, and surgical manipulation [4,5,6]. Mechanical lung injury triggers activation of a proinflammatory cytokine cascade, a process known as biotrauma, which may amplify local pulmonary inflammation and contribute to systemic inflammatory propagation [7,8].

Despite advances in ventilatory techniques, hypoxemia during OLV remains a common intraoperative complication, with varying incidence rates reported in the literature [2]. Predictors remain inconsistent and have limited practical utility, making it difficult to identify at-risk patients. This has led to growing interest in systemic inflammatory markers as potential tools for risk stratification [9]. Several predictors of hypoxemia during OLV have been reported, but accurately identifying patients at risk before surgery remains challenging [10].

In recent years, the neutrophil-to-lymphocyte ratio (NLR) and platelet-to-lymphocyte ratio (PLR) have emerged as simple and inexpensive markers of systemic inflammation and immune dysregulation [11]. Both are derived from complete blood counts and reflect systemic inflammatory response [12].

NLR and PLR have been associated with adverse postoperative outcomes including pulmonary complications, particularly in lung cancer and thoracic surgery cohorts. However, evidence regarding their relationship with intraoperative hypoxemia during OLV is still limited [11,13].

Few studies have examined whether preoperative inflammatory biomarkers can help predict acute intraoperative hypoxemia during one-lung ventilation. Most existing prediction models have focused instead on physiological, demographic, or intraoperative factors. The objective of this study was to evaluate whether preoperative NLR and PLR are associated with severe intraoperative hypoxemia during OLV and whether they improve risk prediction beyond standard clinical variables.

## 2. Materials and Methods

This was an analysis of an ongoing single-center prospective observational cohort. A total of 103 adult patients scheduled for elective thoracic surgery requiring OLV were enrolled between January 2024 and March 2026 at the Department of Anesthesia and Intensive Care of the Târgu Mureș Emergency Clinical County Hospital, Romania. Patients were enrolled consecutively according to predefined inclusion and exclusion criteria. Patient recruitment remains ongoing.

This study was approved by the hospital’s Ethics Committee (approval no 35163/12.01.2024). GDPR requirements were observed, and all collected data were used for research purposes only.

Inclusion criteria were age ≥18 years and elective thoracic surgery requiring intraoperative OLV. Exclusion criteria included incomplete data, emergency or non-standardized surgical/anesthetic management, pre-existing severe respiratory/hemodynamic instability, conditions influencing inflammatory markers (such as active infection, recent corticosteroid or immunosuppressive therapy) and failure to obtain valid ABG during OLV.

All patients received general anesthesia with lung isolation using a double-lumen tube. Anesthesia was maintained with sevoflurane in all patients. Surgical approach was categorized as thoracotomy or thoracoscopy/VATS.

A lung-protective ventilation strategy was applied during OLV. A tidal volume of 6 mL/kg predicted body weight and positive end-expiratory pressure (PEEP) of 5 cmH_2_O were applied to the dependent ventilated lung. The inspired oxygen fraction (FiO_2_) was adjusted intraoperatively according to the patient’s oxygenation status, with the aim of maintaining peripheral oxygen saturation between 92% and 94% whenever clinically feasible. FiO_2_ was increased when necessary to correct clinically significant desaturation, at the discretion of the attending anesthesiologist.

### 2.1. Data Collection

Demographic and clinical data were collected. Preoperative laboratory analyses were obtained on the day of surgery and included a complete blood count from peripheral blood, from which NLR and PLR were calculated. Peripheral oxygen saturation (SpO_2_) was recorded as part of standard monitoring during general anesthesia.

Intraoperative variables included arterial blood gas analyses performed during OLV, OLV duration, total operative time, and duration of postoperative mechanical ventilation. Because ABG sampling was clinically driven, the selected PaO_2_/FiO_2_ represents the lowest documented intraoperative oxygenation value rather than a protocol-fixed time point.

### 2.2. Outcome Definition

The primary outcome was severe intraoperative hypoxemia during OLV, defined as PaO_2_/FiO_2_ ratio < 100 [14]. Although the PaO_2_/FiO_2_ threshold of <100 was originally proposed within the Berlin ARDS definition, it was used in the present study as a marker of severe oxygenation impairment rather than as a diagnostic criterion for ARDS.Statistical analysis.

Data distribution was assessed using the Shapiro–Wilk test. Because most continuous variables were non-normally distributed, continuous data are presented as median and interquartile range (IQR). Categorical variables are presented as absolute and relative frequencies. Comparisons between hypoxemic and non-hypoxemic groups were performed using the Mann–Whitney U test for continuous variables and the chi-square test or Fisher’s exact test for categorical variables, as appropriate.

Hierarchical binary logistic regression models were constructed to evaluate predictors of severe intraoperative hypoxemia and the incremental predictive value of preoperative inflammatory markers.

Model 1 (M1) included age, BMI, preoperative SpO_2_, surgical approach, and OLV duration. Model 2 (M2): added preoperative NLR. Model 3 (M3): added preoperative PLR.

Results are reported as odds ratios (OR/Exp[B]) with 95% CI. Model performance was evaluated using Nagelkerke R^2^, Akaike information criterion (AIC), Bayesian information criterion (BIC), and receiver operating characteristic (ROC) curve analysis. Sensitivity and specificity were calculated using a classification threshold of 0.5.

In addition, an exploratory physiological multivariable regression model including intraoperative gas-exchange parameters was performed to describe variables associated with hypoxemia status. This model was not considered an independent predictive model because several included variables were physiologically or mathematically related to the outcome definition.

A *p*-value < 0.05 was considered statistically significant. Statistical analysis was performed using SPSS version 29.0.

## 3. Results

A total of 103 patients undergoing thoracic surgery with OLV were included in the present interim analysis. Severe intraoperative hypoxemia occurred in 44 patients (42.7%), while 59 (57.3%) patients were classified as non-hypoxemic.

The median age was 61.0 years. Preoperative oxygen saturation (SpO_2_) had a median of 99.0%. The median NLR was 3.39, and the median PLR was 145.16.

Descriptively, patients with hypoxemia had higher BMI and longer OLV duration as well as lower preoperative NLR and PLR values compared with patients without hypoxemia, although none of these differences reached statistical significance. Median NLR was 3.10 in the hypoxemia group versus 3.41 in the non-hypoxemia group, while median PLR was 128.31 versus 175.7, respectively (Table 1).

### Intraoperative Oxygenation During OLV

Age differed significantly between groups in the univariate analysis (Table 1); however, age was not independently associated with severe hypoxemia after adjustment for other variables in the multivariable model. During OLV, patients with hypoxemia had significantly lower SpO_2_, PaO_2_, and PaO_2_/FiO_2_ values, and significantly higher PaCO_2_, Qs/Qt, and A–a gradient compared with non-hypoxemic patients (Table 2).

No significant differences were observed in preoperative inflammatory markers between the two groups. NLR was similar between groups (*p* = 0.568), while PLR showed a non-significant trend toward lower values in the hypoxemia group (*p* = 0.061).

To assess whether preoperative inflammatory biomarkers added predictive value beyond standard clinical variables, three hierarchical logistic regression models were constructed.

The baseline clinical model (M1) was statistically significant compared with the null model (χ^2^ = 16.21, *p* = 0.006; Nagelkerke R^2^ = 0.208). The addition of NLR (M2) did not improve model fit (Δχ^2^ = 0.083, *p* = 0.774; Nagelkerke R^2^ = 0.209). Adding PLR significantly improved model discrimination (M3) (Δχ^2^ = 8.87, *p* = 0.003), increasing the explained variance to 30.9% and yielding the lowest AIC and BIC values (Table 3).

For M1, χ^2^ refers to improvement over the null model. For M2 and M3, Δχ^2^ refers to improvement over the previous model.

In the multivariable analysis, BMI emerged as an independent predictor of hypoxemia (OR 1.12, *p* = 0.009).

In the final model (M3), BMI remained significantly associated with severe intraoperative hypoxemia (OR 1.13, *p* = 0.008). Preoperative PLR was inversely correlated with severe intraoperative hypoxemia; expressed per 10-unit increase, higher PLR was associated with lower odds of hypoxemia (OR 0.895, 95% CI 0.817–0.980, *p* = 0.016). NLR was not associated with the outcome. Thoracotomy was associated with higher odds of hypoxemia compared with thoracoscopy (OR 2.29), although this did not reach statistical significance (*p* = 0.171). OLV duration was not significantly associated with hypoxemia when expressed per 10-min increase (OR 1.02, 95% CI 0.94–1.10, *p* = 0.584) (Table 4).

**Figure 1:**
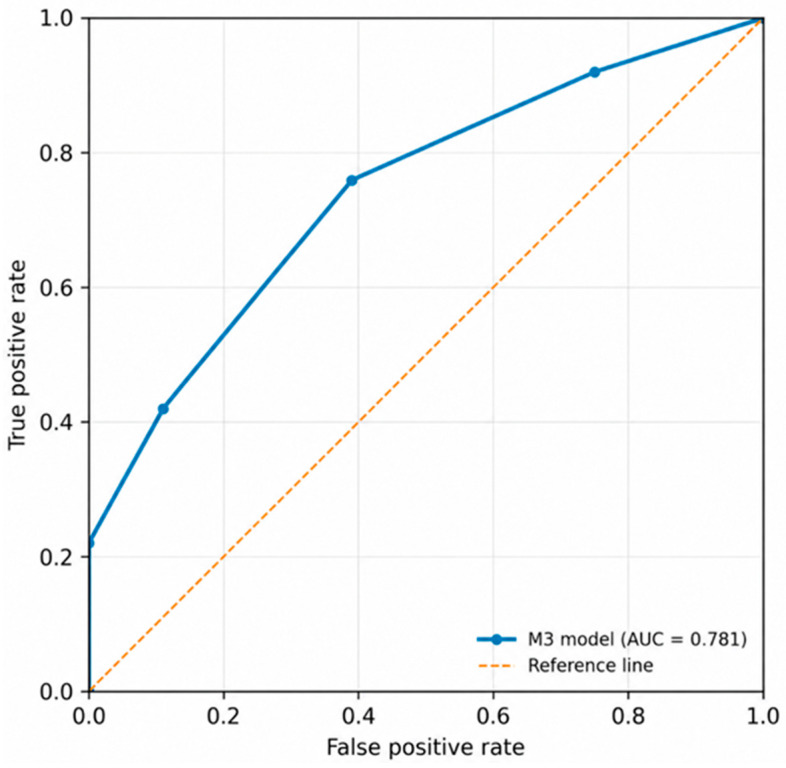
ROC curve for the final hierarchical logistic regression model (M3) for prediction of severe intraoperative hypoxemia during OLV. AUC = 0.781, 95% CI 0.688–0.874.

The final model (M3) showed acceptable discrimination (AUC = 0.781, 95% CI 0.688–0.874). At a cut-off value of 0.5, the specificity was 79.2%, and the sensitivity was 62.8% (Figure 1). Using the Youden index, the optimal probability threshold was 0.648, corresponding to a sensitivity of 83.3% and a specificity of 61.8%.

An exploratory physiological regression model was performed to describe variables associated with hypoxemia status after inclusion of intraoperative gas-exchange parameters. In this model, preoperative PLR, PaO_2_, Qs/Qt, and A–a gradient were significantly associated with hypoxemia status (Supplementary Table S1). Because several included variables are physiologically or mathematically related to the outcome definition, this model should not be interpreted as an independent predictive model. Results of the exploratory physiological regression analysis are presented in Supplementary Table S1.

## 4. Discussion

This study evaluated the relationship between preoperative inflammatory markers and intraoperative hypoxemia during OLV. The main finding was that BMI was the most consistent clinical predictor of severe intraoperative hypoxemia, while preoperative PLR improved the performance of the clinical model. In contrast, NLR was not associated with hypoxemia and did not improve model performance. Given the clinical relevance of hypoxemia during OLV and its potential impact on intraoperative management, identifying patients at increased risk remains an important objective in thoracic anesthesia [15].

Our findings differ from previous studies evaluating inflammatory ratios in lung cancer surgery. Lan et al. reported that elevated preoperative NLR and PLR were associated with postoperative pulmonary complications after radical lung cancer surgery, while Xiaowei et al. reported similar associations for inflammatory hematological parameters following lung cancer resection [16,17]. Wang et al. also showed that postoperative increases in NLR and PLR were associated with postoperative pulmonary complications in patients with non-small cell lung cancer [11]. However, these studies mainly evaluated postoperative pulmonary complications, whereas the endpoint of the present study was acute intraoperative hypoxemia during OLV. This difference in timing and outcome definition may explain why NLR was not predictive in our cohort.

The observed inverse association between PLR and hypoxemia suggests that PLR may reflect mechanisms beyond a simple systemic inflammatory response. Instead, it may reflect a more complex platelet-lymphocyte balance. Nevertheless, this finding requires further investigation. Although the biological explanation remains uncertain, platelets may play a role in pulmonary vascular regulation and inflammatory responses. Platelets are increasingly recognized as active participants in pulmonary vascular and endothelial responses [18]. Experimental and clinical evidence suggests that platelet activation may influence endothelial function, pulmonary vascular remodeling, and inflammatory signaling through interactions with leukocytes and the vascular endothelium. Therefore, PLR may reflect a combination of immune and vascular processes rather than systemic inflammation alone [18,19]. However, these mechanisms were not directly evaluated in the present study, and the observed inverse association should be interpreted cautiously and considered hypothesis-generating. Platelet-related inflammatory and microvascular responses during OLV may contribute to impaired oxygenation, although this hypothesis requires further validation [20,21].

BMI emerged as the most consistent clinical factor associated with hypoxemia in our analysis. This association may be explained by reduced functional residual capacity and respiratory system compliance associated with increased BMI, which may promotes atelectasis and increase V/Q mismatch during OLV. These mechanisms may reduce oxygenation reserve during one-lung ventilation and increase susceptibility to severe intraoperative desaturation [22]. Although age differed significantly between groups in the unadjusted comparison, it was not independently associated with severe intraoperative hypoxemia in the final multivariable model. Surgical approach did not independently explain hypoxemia in our cohort. Although thoracotomy showed higher odds of intraoperative hypoxemia, this association did not reach statistical significance (OR = 2.292, *p* = 0.171) and should therefore be interpreted only as a trend. Previous studies generally reported better perioperative outcomes and fewer postoperative complications after VATS compared with thoracotomy. However, these outcomes are predominantly postoperative and do not necessarily translate into an independent effect on acute intraoperative hypoxemia, which is consistent with our findings [23].

In our cohort, NLR was not associated with severe intraoperative hypoxemia during OLV, suggesting that neutrophil-dominant systemic inflammation may be less relevant to the acute onset of oxygenation impairment during OLV. This may explain why preoperative NLR did not improve prediction in our cohort, as it may be less informative for acute intraoperative oxygenation impairment than for broader postoperative inflammatory outcomes.

Clinically, preoperative hematological indices may provide complementary information for risk stratification, but their clinical utility remains uncertain. Although PLR improved the statistical performance of the model, sensitivity remained moderate, indicating that a relevant proportion of patients with severe hypoxemia would not be identified. Therefore, these markers should not replace established clinical assessment, intraoperative monitoring, or physiological predictors.

This study has several limitations. First, this was an interim analysis of an ongoing single-center prospective observational cohort with a relatively small sample size and a limited number of hypoxemia events. The relatively low events-per-variable ratio may have increased the risk of model overfitting and unstable coefficient estimates. As this is an interim analysis of an ongoing cohort, the findings should be considered preliminary. Therefore, the multivariable regression models should be interpreted as exploratory and require validation in larger independent cohorts. Second, arterial blood gas sampling during OLV was performed according to clinical sign rather than at a fixed protocol-defined time point. Therefore, the lowest documented PaO_2_/FiO_2_ value may have been influenced by sampling frequency and clinical decision-making. In addition, FiO_2_ was titrated intraoperatively according to oxygenation targets and clinical judgment. Because PaO_2_/FiO_2_ is influenced by FiO_2_ adjustment, the outcome reflects real-world intraoperative management rather than oxygenation measured under a fixed FiO_2_ protocol. This limitation should be considered when interpreting the observed association between PLR and severe intraoperative hypoxemia.

The observed inverse relationship between PLR and hypoxemia should also be interpreted cautiously. PLR may be influenced by oncological status, nutritional state, smoking, medication, subclinical inflammatory conditions, and other unmeasured confounders. Moreover, platelet activation, endothelial dysfunction, cytokine release, and microvascular responses were not directly assessed.

Finally, the exploratory physiological regression model included gas-exchange variables that are closely related to the outcome definition, including PaO_2_, Qs/Qt, and the A–a gradient. For this reason, the model should be viewed as a descriptive physiological analysis rather than an independent predictive model.

## 5. Conclusions

In our study, BMI was the most consistent clinical predictor of severe intraoperative hypoxemia. Preoperative NLR was not associated with hypoxemia and did not improve model performance. PLR improved the predictive performance of the clinical model, but its association was inverse and should be interpreted as exploratory. Our findings suggest that preoperative hematological indices may provide complementary information for risk stratification, but they require confirmation in larger cohorts with standardized intraoperative sampling and external validation.

## Figures and Tables

**Table 1:** Descriptive statistics.

Variable	Total Cohort n = 103	Non-Hypoxemia n = 59 (57.28%)	Hypoxemia n = 44 (42.71%)	*p*-Value
Male sex, n (%)	66 (64.1%)	38 (64.4%)	28 (63.6%)	0.936
Thoracotomy, n (%)	77 (74.8%)	42 (71.2%)	35 (79.5%)	0.334
ARISCAT high risk, n (%)	58 (56.3%)	32 (54.2%)	26 (59.1%)	0.623
Age, years	61.0 (53–68)	66.0 (52–70.5)	58.0 (53–63.5)	0.026
BMI, kg/m^2^	25.06 (22.13–28.61)	25.01 (21.8–26.76)	25.46 (22.61–32.28)	0.078
ASA score	3.0 (3–4)	3.0 (3–4)	3.0 (3–4)	0.159
Preoperative SpO_2_, %	99.0 (98–100)	99.0 (98–100)	99.0 (98–100)	0.364
Preoperative NLR	3.39 (2.26–4.8)	3.41 (2.45–4.9)	3.10 (2.02–4.78)	0.568
Preoperative PLR	145.16 (98.99–200.81)	175.7 (103.12–202.25)	128.31 (92.52–190.09)	0.061
OLV duration, min	130.0 (70–180)	120.0 (62.5–150)	145.0 (82.5–180)	0.126
Surgery duration, min	170.0 (120–210)	160.0 (115–200)	180.0 (120–222.5)	0.333
ARISCAT score	50.0 (40–51)	47.0 (40–54)	50.0 (43–51)	0.954
SPORC score	7.0 (6–8)	7.0 (5–8)	8.0 (6–8)	0.184

**Table 2:** Intraoperative oxygenation parameters during OLV according to hypoxemia status.

Variable	Non-Hypoxemia n = 59	Hypoxemia n = 44	*p*-Value
SpO_2_ OLV, %	97 (94–99)	90 (88–95)	<0.001
FiO_2_ OLV, %	95 (70–100)	100 (80–100)	0.039
PaO_2_ OLV, mmHg	105 (92–156)	66.5 (56–78)	<0.001
PaCO_2_ OLV, mmHg	45 (41–50)	53 (45–63)	<0.001
PaO_2_/FiO_2_ OLV	156 (130–190)	70.5 (56–80)	<0.001
Qs/Qt OLV, %	30 (27–34)	47 (42–57)	<0.001
A–a gradient OLV, mmHg	354 (283–419)	576 (513–585)	<0.001

**Table 3:** Incremental performance of hierarchical logistic regression.

Model	Variables Included	χ^2^/Δχ^2^	*p*-Value	Nagelkerke R^2^	AIC	BIC
M1	Age, BMI, preoperative SpO_2_, surgical approach, OLV duration	16.21	0.006	0.208	127.84	143.22
M2	M1 + NLR	0.083	0.774	0.209	129.75	147.70
M3	M2 + PLR	8.87	0.003	0.309	122.88	143.40

**Table 4:** Final hierarchical logistic regression model for predicting severe intraoperative hypoxemia.

Variable	OR/Exp(B)	95% CI	*p*-Value
Thoracotomy	2.29	0.70–7.53	0.171
Age	0.97	0.93–1.01	0.089
BMI	1.13	1.03–1.23	0.008
Preoperative SpO_2_	0.98	0.89–1.07	0.642
OLV duration/10 min	1.02	0.99–1.10	0.584
Preoperative NLR	1.25	0.94–1.69	0.143
Preop PLR/10 units	0.895	0.817–0.980	0.016

Note: OR for PLR is reported per 10-unit increase.

## Data Availability

The data used for this study can be found in the database of the Târgu Mures, County Emergency Clinical Hospital, Romania.

## Supplementary Materials

The following supporting information can be downloaded at: https://www.mdpi.com/article/10.3390/life16071057/s1, Table S1: Exploratory physiological regression analysis adjusted for intraoperative gas-exchange parameters.

## Abbreviations

The following abbreviations are used in this manuscript:

Abbreviation	Definition
A–a gradient	Alveolar–arterial oxygen gradient
ABG	Arterial blood gas
AIC	Akaike information criterion
ASA	American Society of Anesthesiologists
AUC	Area under the curve
BIC	Bayesian information criterion
BMI	Body mass index
CI	Confidence interval
FiO_2_	Fraction of inspired oxygen
IQR	Interquartile range
NLR	Neutrophil-to-lymphocyte ratio
OLV	One-lung ventilation
OR	Odds ratio
PaCO_2_	Partial pressure of arterial carbon dioxide
PaO_2_	Partial pressure of arterial oxygen
PaO_2_/FiO_2_	Ratio of arterial oxygen partial pressure to inspired oxygen fraction
PEEP	Positive end-expiratory pressure
PLR	Platelet-to-lymphocyte ratio
Qs/Qt	Pulmonary shunt fraction
ROC	Receiver operating characteristic
SpO_2_	Peripheral oxygen saturation
SPSS	Statistical Package for the Social Sciences
V/Q mismatch	Ventilation–perfusion mismatch
VATS	Video-assisted thoracic surgery
