# Supplementary material for: Preoperative Inflammatory Ratios and Severe Intraoperative Hypoxemia During One-Lung Ventilation: A Prospective Observational Study
Source: Life (Basel). 2026 Jun 25;16(7):1057. doi: 10.3390/life16071057 (PMC13412962; doi:10.3390/life16071057)
Supplement: Supplementary file 1 [file life-16-01057-s001.zip › life-4383827-supplementary.pdf]

Supplementary Table S1. Exploratory physiological regression analysis adjusted for intraoperative gas-exchange parameters.

Variable	Unstandardized B	Standard error	Standardized β	p-value	VIF
Preoperative PLR	−0.001	0.000	−0.166	0.044	1.931
Intraoperative PaO_2	−0.003	0.001	−0.265	0.002	2.062
Intraoperative Qs/Qt	0.012	0.005	0.267	0.014	3.302
Intraoperative A–a gradient	0.002	0.000	0.369	<0.001	3.299

Model summary: $R = 0.861$; $R^2 = 0.741$; adjusted $R^2 = 0.690$; $p < 0.001$.

Note: Only variables with $p < 0.05$ are shown. The respiratory index was excluded due to collinearity (VIF > 10).
